# Human microglia differentially respond to β‐amyloid, tau, and combined Alzheimer's disease pathologies in vivo

**DOI:** 10.1002/alz.70930

**Published:** 2025-11-21

**Authors:** Morgan A. Coburn, Ghazaleh Eskandari‐Sedighi, Jonathan Hasselmann, Whitney England, Sepideh Kiani Shabestari, Kimiya Mansour, Amanda McQuade, Michael Armen Mgerian, Jean Paul Chadarevian, Christina Tu, Jorge Silva, Jaclyn Beck, Zahara Keulen, Robert C. Spitale, Hayk Davtyan, Mathew Blurton‐Jones

**Affiliations:** ^1^ Department of Neurobiology and Behavior University of California Irvine California USA; ^2^ Sue and Bill Gross Stem Cell Research Center University of California Irvine California USA; ^3^ Institute for Memory Impairments and Neurological Disorders University of California Irvine California USA; ^4^ Center for the Neurobiology of Learning and Memory University of California Irvine California USA; ^5^ Department of Pharmaceutical Sciences University of California Irvine California USA

**Keywords:** Alzheimer's disease, amyloid beta, human microglia, iPSC, rod microglia, tau, type I interferon

## Abstract

**INTRODUCTION:**

Recent studies have identified important species‐dependent differences in the response of microglia to β‐amyloid (Aβ) pathology. Yet, whether human microglia also interact differently with the pathognomonic combination of amyloid and tau pathologies that occur in Alzheimer's disease (AD) remains unclear.

**METHODS:**

We generated a xenotolerant mouse model of AD that develops both plaque and tangle pathologies, transplanted stem cell‐derived microglial progenitors and examined the interactions between human microglia and AD pathologies with scRNA sequencing, immunohistochemistry, and in vitro modeling.

**RESULTS:**

The combined amyloid and tau pathologies induced robust type‐I interferon and proinflammatory cytokine responses, as well as an increased adoption of a distinct “rod” morphology in human microglia. The rod morphology could be induced with type‐I interferon treatment in vitro.

**DISCUSSION:**

We provide new insights into human microglial responses to combined AD pathologies and a novel platform to investigate and manipulate human microglia in vivo.

**Highlights:**

- Amyloid pathology promotes the rapid development of neurofibrillary tangles and neuronal loss in a novel chimeric model of AD.
- Combined Alzheimer's disease pathologies lead to an expansion of disease‐associated microglia (DAM) and exacerbate Interferon‐responsive and cytokine/chemokine‐enriched states in xenotransplanted human microglia.
- The combination of amyloid and tau promotes the development of a distinctive rod microglial phenotype that closely correlates with tau pathology and neurodegeneration.
- Rod morphology and transcriptional changes can be modeled in vitro by treatment of induced pluripotent stem cells (iPSC) ‐microglia with type‐I interferons.

## 1 BACKGROUND

Alzheimer's disease (AD) is a progressive neurodegenerative disease characterized pathologically by the accumulation of amyloid‐β (Aβ) plaques and neurofibrillary tangles composed of hyperphosphorylated tau. Genome‐wide association studies (GWAS) have shown that late‐onset AD risk is associated with polymorphisms in over 80 loci, the majority of which are associated with genes that are highly or uniquely expressed in microglia.^1^, ^2^, ^3^ While most of these genes confer relatively small individual effects, together they combine to drive 60%–80% of total AD risk.^4^, ^5^ Despite immense progress in the identification of microglial‐enriched AD risk genes, our collective understanding of their functional consequences remains limited, in part because homology of AD risk genes between humans and mice is often modest.^6^ Thus, studies that examine how human microglia respond to and interact with Aβ plaques and tau‐laden neurofibrillary tangles could offer important new insight.

Microglia are highly responsive to their local environment and alter their transcriptomic and functional responses to varying pathologies, injuries, and culture conditions.^7^, ^8^, ^9^, ^10^, ^11^ Given this remarkable environmental sensitivity, we previously developed a chimeric mouse model that introduces human microglia progenitors into the brains of postnatal, immunodeficient, xenotolerant mice.^7^ This model achieves up to 80% human microglial chimerism within the forebrain and has enabled in vivo transcriptional and functional analyses of human microglia and examinations of the impact of AD risk genes on microglial responses to Aβ plaque pathology.^7^, ^12^, ^13^ Given the genetic divergence between many human and mouse AD risk genes, including *CD33*, *CR1*, apolipoprotein E (*APOE*), *HLA‐DRB1*, *MEF2C*, and *OAS1*, this chimeric model further serves as an important approach to assess the uniquely human responses of microglia to AD pathologies.^6^, ^14^, ^15^

The transcriptional and physical interaction of murine microglia with Aβ plaques have been well‐established, including the adoption of a disease‐associated microglia (DAM) state.^16^, ^17^, ^18^ Similarly, numerous studies have explored the murine microglia responses to tau pathology, although this has resulted in some discrepancies on the impact of microglia on disease progression.^19^, ^20^, ^21^ In contrast, our understanding of human microglial responses to combined amyloid and tau pathologies in vivo have largely been restricted to *post mortem*, end‐stage analyses.^22^, ^23^ To further understand how amyloid and tau pathologies influence human microglia in vivo and to provide a tractable system to study and manipulate these interactions, we have now generated and examined two novel chimeric mouse models. Taken together, we provide evidence that amyloid and tau pathology independently promote varying microglial activation states, but that the combination of these hallmark pathologies promotes a robust type I interferon response and the development of a highly distinct microglial phenotype.

## 2 METHODS

### 2.1 Animals

All animal procedures were conducted in accordance with the guidelines set forth by the National Institutes of Health and the University of California, Irvine Institutional Animal Care and Use Committee. The “hCSF1” knock‐in mouse model was purchased from Jackson Laboratories (stock #017708) and includes deletions of Rag2^−/−^ and Il2rγ^−/y^, and humanized CSF1. “hPS19 mice” were generated by backcrossing PS19 mice (JAX stock #008169) onto the hCSF1 background and restoring homozygosity for hCSF1, Rag2, and Il2rγ alleles while maintaining hemizygosity for the PS19 tau transgene. While the P301S tau mutation leads to frontotemporal dementia (FTD), not AD, these mice develop neurofibrillary tangle pathology that closely mimics that of AD patients and are therefore commonly used to examine both FTD‐ and AD‐related aspects of tau pathology.^19^, ^24^, ^25^ The “h5XfAD” model was previously generated using a similar strategy of backcrossing 5XFAD mice with hCSF1 mice.^12^ To generate all four genotypes examined in the current study (hWT, h5XFAD, hPS19, and hPS‐5X), h5XFAD mice were crossed with hPS19 mice. All groups were age and sex‐matched and group housed on a 12 h/12 h light/dark cycle with food and water ad libitum.

### 2.2 Acquisition and maintenance of induced pluripotent stem cell lines

A cytoplasmic green fluorescent protein (GFP) ‐expressing induced pluripotent stem cell (iPSC) line (Coriell, AICS‐0036) was purchased from Coriell and previously generated by CRISPR modification of the line WTC11 to insert a monoallelic monomeric enhanced GFP (mEGFP) into the AAVS1 safe‐harbor locus under a CAG promoter. The iPSC lines were maintained by culturing in feeder‐free conditions in complete TeSR E8 medium (StemCell Technologies, Inc.), in a humidified incubator (5% CO_2_, 37°C), with medium changed daily. Passaging was performed every 7–8 days using ReLeSR (StemCell Technologies, Inc.) and cells were plated onto six‐well plates (Corning), coated with growth‐factor‐reduced Matrigel (1 mg/plate; BD Biosciences), in TeSR E8 medium, supplemented with 0.5 µM thiazovivin (StemCell Technologies, Inc.) for the first 24 h post‐passage. All iPSC lines used were confirmed to be negative for mycoplasma and exhibit a normal karyotype.

**RESEARCH IN CONTEXT**

1. **Systematic review**: We reviewed the literature using available datasets and resources (e.g., PubMed).
2. **Interpretation**: Using a novel chimeric model of Alzheimer's disease (AD), we find that human microglia exhibit differing responses to amyloid and tau pathology. In addition, we find that the combination of both hallmark pathologies promotes an interferon responsive signature in transplanted human microglia and increased adoption of a distinct rod microglial phenotype.
3. **Future directions**: Our xenotolerant mouse model with combined amyloid and tau pathologies provides a promising new approach to manipulate and study the interactions between human microglia and AD pathologies in vivo. This unique model can be used to further decipher the mechanistic links between amyloid and tau pathologies and the potential role of microglia in AD‐related neurodegeneration. This platform can also be used to examine the mechanisms through which genetic variants modulate the response of human microglia to amyloid and tau pathologies.

### 2.3 Differentiation of hematopoietic progenitor cells and microglia from iPSCs

iPSCs were differentiated into induced hematopoietic progenitor cells (iHPCs) and human iPSC‐derived microglia (iMG) following our previously published protocol.^26^ Briefly, iPSCs were passaged in mTeSR1 to achieve a density of 40 colonies of ∼100 cells each per 35 mm well on Matrigel‐coated plates (1 mg/plate). On day 0, cells were transferred to Medium A from the STEMdiff Hematopoietic Kit (Stem Cell Technologies Cat. #05310). On day 3, cells were exposed to Medium B and remained in Medium B for 7 additional days while small round iHPCs began to lift off the colonies. On days 10–12, medium and floating cells were carefully removed with a serological pipette to collect non‐adherent CD43+ iHPCs. At this point, days 10–12 iHPCs were frozen in Bambanker (Wako) to avoid batch differences throughout the study. Cells used for early‐postnatal iHPC transplantation were thawed into iMG medium (Dulbecco's modified Eagle's medium [DMEM]/F12, 2× insulin‐transferrin‐selenite, 2× B27, 0.5× N2, 1× GlutaMAX, 1× non‐essential amino acids, 400 mM monothioglycerol, and 5 mg/mL human insulin) freshly supplemented with 100 ng/mL interleukin [IL]‐34, 50 ng/mL transforming growth factor beta 1 (TGFβ1), and 25 ng/mL M‐cerebrospinal fluid (M‐CSF; Peprotech) and allowed to recover for 24–48 h, then resuspended at 62,500 cells/µL in 1× Dulbecco's phosphate buffered saline (DPBS; low Ca^2+^, low Mg^2+^). To continue differentiation of iHPCs to iMG for in vitro studies, media was added to thawed or fresh iHPCs every 48 h for 28 days with extra cytokines CD200 (100 ng/mL, Novoprotein) and CXCL1 (100 ng/mL; PeproTech) added for the last 3 days in culture.

### 2.4 Treatment of in vitro microglia and IncuCyte imaging

Mature iMG were plated at 30% confluence onto fibronectin‐coated glass imaging plates (StemCell Technologies, Corning). Cells were allowed to settle 1 h and then treated for 48 h with IFNα (100 ng/mL), IFNβ (100 ng/mL), a combination of interferon (IFN) α/β (50 ng/mL each), IFNγ (100 ng/mL), or IL‐1β (100 ng/mL). Inflammasome activation followed a classical lipopolysaccharide+ adenosine triphosphate (LPS+ATP) paradigm; 100 ng/mL LPS for 3 h, followed by addition of 10 µM ATP. Phase‐contrast images and corresponding cell masks were collected from seven wells per treatment using an Incucyte S3 live‐cell imaging system.

### 2.5 Early postnatal intracerebroventricular transplantation of microglial progenitors

P0 mouse pups were tail‐clipped for overnight polymerase chain reaction (PCR) to probe for sex, hPS19, and h5XFAD genotypes and tattooed for later identification. For 1–2 days later, pups with each of the four genotypes received bilateral intracerebroventricular transplants of 500,000 total iPSC‐derived HPCs according to our previously detailed methods.^7^ In briefly, pups were placed in a clean cage over a heating pad with a nestlet from the home cage to maintain the mother's scent. Pups were then placed on ice for 2–3 min to induce hypothermic anesthesia. Free‐hand transplantation was performed using a 30‐gauge needle affixed to a 10 mL Hamilton syringe, mice received 1 µL of iPSC‐derived HPCs suspended in sterile 1× DPBS at 62,500 cells/mL at each injection site (eight sites) totaling 500,000 cells per pup. Bilateral injections are performed at 2/5th of the distance from the lambda suture to each eye, injecting into the lateral ventricles 3 mm down from the surface of the skull and into the overlying anterior cortex at 1 mm down, and into the posterior cortex in line with the forebrain injection sites, and perpendicular to lambda at a 45° angle. Transplanted pups are then returned to their home cages and weaned at P21.

### 2.6 Immunohistochemistry, immunocytochemistry, and confocal microscopy

At 6 months of age, mice were administered Euthasol and monitored for loss of consciousness and toe‐pinch response. Mice were then intracardially perfused with ice cold 1× DPBS and brains hemisected along the mid‐sagittal plane. Half‐brains were then drop fixed in 4% (w/v) PFA for 48 h. Samples were then cryoprotected in 30% (w/v) sucrose and then cut coronally at 40 µm on a freezing sliding microtome. Tissue sections were collected and stored as free‐floating sections in phosphate buffered saline (PBS) with 0.05% sodium azide. For staining, tissue was blocked for 1 h in 1× PBS, 0.2% Triton X‐100, and 10% goat or donkey serum. For Aβ plaque visualization sections were stained with Amylo‐Glo (1:100; Biosensis, Cat. #TR‐300‐AG) for 20 min. Immediately following blocking and Amylo‐Glo staining if applicable, sections were placed in primary antibodies diluted in 1× PBS and 1% goat or donkey serum and incubated overnight on a shaker at 4°C. Primary antibodies used include: eGFP (1:500; Millipore, Cat. #Ab16901), HT7 (1:1000; Thermo Fisher), 82E1 (1:55; Immunobiological labs), AT8 (1:1000; Invitrogen), NeuN (1:1000; Millipore), MX1 (1:200; Cell Signaling, Cat. #37849), CD9 (1:200; Biolegend, Cat. #312102), and HLA‐DR (1:200; Invitrogen, Cat. #14‐9956‐82). Following overnight primary antibody incubation sections were placed in appropriate Alexa Fluor conjugated secondary antibodies (Life Technologies) for 1 h, before rinsing and mounting on microscope slides with Fluoromount G (Southern Biotech). For the in vitro studies, iMG were fixed with pre‐warmed 4% PFA for 7 min and washed 2× with DPBS. For immunocytochemistry, cells were blocked for 1 h in 1× PBS, 0.2% Triton X‐100, and 10% goat or donkey serum then incubated in primary antibodies diluted in 1× PBS and 1% goat or donkey serum and for 1 h at room temp or overnight at 4°C. Cells were then incubated in Alexa‐conjugated secondary antibodies for 1 h, before washing and bathing in PBS for imaging. Immunofluorescent brain sections and fixed iMG were then imaged using an Olympus FX3000 confocal microscope. Images collected for quantitative analysis of amyloid and tangle pathologies were captured at 10× by a blinded observer using identical parameters for a given analysis. Images for quantification were captured from CA1 of the dorsal hippocampus and from the posterior parietal association area (layer 6) located above the corpus callosum and directly superior to the imaged CA1 field. For IMARIS‐based analysis of MX1 and HLA‐DR in rod iMG, eight single z‐plane images were taken across two wells at 40×. For some representative images brightness and contrast settings were slightly adjusted to reveal fine structures and morphology and pseudocolored for clarity and consistency. Such adjustments were only made to non‐quantified representative images and applied equally across groups.

### 2.7 IMARIS quantification and statistical analysis

All quantification of confocal images was performed blinded to genotype or treatment. To examine human microglial interactions with Aβ plaques and neurofibrillary tangles, sections were stained with antibodies against GFP (to amplify endogenous expression in xenografted human microglia [xMG]), Amylo‐Glo (amyloid plaques), 82E1 (anti‐Aβ antibody), and AT8 (phosphorylated tau tangles). IMARIS (version 9.7.0) ‐based quantification of Aβ plaques and tau pathology was performed using the “Volume” function to provide information on z‐stacked volumes. To assess in vivo microglial elongation, z‐stacks were first processed using a median filter in order to better define edges and subtract autofluorescence, then the “Surfaces” function was used to define GFP‐positive cells and Object‐Oriented Bounding Box Lengths A, B, and C were assessed. Total plaque volume (h5XFAD vs. hPS‐5X) and AT8 area (hPS19 vs. hPS‐5X) were tested for statistical significance (*p* < 0.05) through unpaired *t*‐tests. To assess the total length of iMG in vitro, the ImageJ plugin, FiloQuant, was used.^27^ The iMG length were tested for statistical significance (*p* < 0.05) through two‐way ANOVA with multiple comparisons using Prism 9. The CD9 and MX1 expression, microglial elongation, MX1, and CD9 volume and intensity were tested for statistical significance (*p* < 0.05) between all four groups by one‐way analysis of variance (ANOVA) with Tukey's multiple comparisons using Prism 10. To quantify the total volume of xMG relative to plaques (Amylo‐Glo) and tangles (AT8) from confocal z‐stack images, we utilized Bitplane IMARIS (version 10). For neuronal counts we used the “Dot/Points” method to quantify the NeuN positive neurons in cortex and CA1 and tested for statistical significance (*p* < 0.05) by one‐way ANOVA with Tukey's multiple comparisons.

### 2.8 Tissue dissociation and magnetic isolation of xMG for scRNA‐seq

*N* = 4, 6‐month‐old male mice per genotype were euthanized using Euthasol and perfused with ice‐cold PBS, half brains were immediately dissected, the cerebellum removed, and the tissue stored briefly in RPMI. Tissue dissociation was then performed using a Dounce homogenizer. Briefly, each half‐brain was added to a 7 mL Dounce homogenizer containing 4 mL of RPMI, tissue was homogenized by first performing 10 strokes using the “A” pestle, followed by another 10 strokes using the “B” pestle. Homogenate was then passed through a sterile 70 mm filter and pelleted by centrifugation (10 min, 400 × g, 4°C). Myelin and debris were removed by resuspending the pellet in 8 mL 30% Percoll, overlaid with 2 mL of 1× DPBS, spinning at 400 × g for 25 min at 4°C, with acceleration and brake set to 0, and discarding the myelin band and supernatant. Dissociated cell pellets were resuspended in 80 µL fluorescence‐activated cell sorting (FACS) buffer (0.5% BSA in 1× DPBS) + 20 µL Mouse cell removal beads (Miltenyi) to remove all mouse cells and incubated at 4°C for 15 min. Samples were then separated using LS columns and the MidiMACs separator (Miltenyi), and the human cells are collected in the flow‐through. Purified human cells were then pelleted via centrifugation (10 min, 400 × g, 4°C) and dead/apoptotic cells were removed using the EasySep Dead Cell Removal (Annexin V) kit (Miltenyi; Cat. #17899). Cell pellets were resuspended in 70 µL of Dead Cell Removal (DCR) buffer (2% BSA and 1 mM CaCl_2_ in PBS) followed by the addition of 5 µL of the DCR Cocktail and 5 µL of the Biotin Selection Cocktail. Samples were then incubated at room temperature for 3 min. After incubation, 10 mL of DCR beads were added immediately followed by 2.4 mL of DCR buffer and incubated at RT for 10 min on the EasyEights EasySep magnet (StemCell Technologies; Cat. #18103). Keeping the sample on the magnet, the supernatant was collected and pelleted by centrifugation (10 min, 400 × g, 4°C). Cells were then counted on a hemocytometer and resuspended at ∼1000 total cells/µL in FACS buffer. High‐quality and low‐debris samples were then pooled to result in one to two single‐cell RNA‐sequencing (scRNA‐seq) samples per genotype. Two hPS‐5X samples were excluded due to high debris content, and one h5XFAD sample was excluded due to low cell viability. In some cases, two mice per genotype were pooled prior to 10×.

### 2.9 Single‐cell sequencing via 10× Genomics

scRNA‐seq library preparation was performed according to the 10× Genomics Chromium Single Cell 3′ Reagents Kit v3, user guide except that sample volumes containing 25,000 cells are loaded onto the 10× Genomics flow cell to capture ∼10,000 total cells. The 10× Genomics workflow was then followed according to the manufacturer protocol and libraries are pooled at equimolar concentrations for sequencing on an Illumina NovaSeq 6000, targeting ∼50,000 reads per cell. FASTQ files are aligned to the human GRCh38 transcriptome using the Cell Ranger (version 3.0.2) count command, with the expected cells set to 10,000 and no secondary analysis performed.^28^

### 2.10 scRNA‐seq data visualization and differential gene analysis

UMI count tables were read into Seurat (version 3) ^29^ for preprocessing and clustering analysis. Initial quality control (QC) was performed by log‐normalizing and scaling (default settings) each dataset followed by principal component analysis (PCA) performed using all genes in the dataset. Seurat's “ElbowPlot” function was used to select principal components (PCs) to be used for clustering along with a resolution parameter of 0.5 and clusters identified as being doublets, gene‐poor, or dividing are removed from the dataset prior to downstream analysis. Secondary QC cutoffs were then applied to retain only cells with < 20%–25% ribosomal genes, 12.5% mitochondrial genes, > 500 genes but less than double the median gene count, and > 500 UMI but less than double the median UMI count. At this point one hPS19 and one hPS‐5X sample were discarded due to abnormal 10× cell capture and transcript amplification. The final samples that yielded clean and high viability isolates and passed QC are as follows: four hWT mice (pooled two per 10× run into two 10× runs); three h5XFAD mice (two pooled into one 10× run, one in a 10× run independently); two hPS19 mice pooled into one 10× run; one hPS‐5X mouse in one 10× run. Cells passing QC for each sample were then merged using Seurat's “merge” function and datasets were processed using Seurat's integrated analysis workflow.^30^ Briefly, samples from individual mice were integrated using the “FindIntegationAnchors” and “IntegrateData” commands using dimensions 1:25. Datasets were then scaled, and sources of technical variation are regressed out (number of genes, percent ribosomal genes, and percent mitochondrial genes) and PCA was performed using Seurat's “RunPCA” command. A shared nearest neighbor (SNN) plot was generated using Seurat's “FindNeighbors” function using PCs 1:40 as input, clustering was performed using the “FindClusters” function and a resolution parameter of 0.3, and dimension reduction was performed using the “RunUMAP” function with the same PCs used for generating the SNN plot. Differentially expressed genes (DEGs) were determined between clusters using the “FindAllMarkers” function, which employs a Wilcoxon rank sum test, with and FDR cutoff of 0.01, an LFC cutoff of 0.25, and the requirement that the gene be expressed in at least 10% of the cluster and clusters are labeled according to manual curation of the differential gene lists.

### 2.11 Differential proportion analysis (DPA)

To assess potential differences in cluster proportions between genotypes, we utilized a modified version of single‐cell differential composition (scDC) analysis.^31^ Briefly, we used stratified bootstrap resampling as described in Cao et al. to generate additional imputed populations. We initially attempted to re‐cluster each bootstrapped population using Seurat's *FindNeighbors()* and *FindClusters()*.^32^ However, our original clustering contains one very large cluster and multiple clusters that are at least 10 times smaller in cell count. This, in addition to the heterogeneity of the population as a whole, made it difficult for *FindClusters()* to fully partition out all cell types in the bootstrapped samples even with a high‐resolution parameter. Therefore, we opted to instead utilize the alternative *scDC_NoClustering()* library method developed by Cao et al. This method performs bootstrap resampling a total of 10,000 times, and calculates new proportions and cell counts from the original cluster identities. We then fit a generalized linear model (GLM) to each set of imputed cell populations using the following formula: glm(cellCount ∼ cellType*genotype, family = poisson(link = *log))* where *cellCount* is the number of cells identified as a certain cell type for a certain genotype. Before fitting the model, total cell counts were normalized across samples and genotypes, while maintaining the proportion of cells for each cell type within each sample. Statistics from each GLM were pooled as in Cao et al. using the R package *mice*’s *pool()* function.^33^ Significance of the pooled estimates was determined by the univariate Wald test. As we found significant interactions between multiple cell types and genotypes, we also conducted post‐hoc pairwise comparisons. For each GLM, we ran the *multcomp* function *glht(fit, linfct =* *emm(pairwise∼genotype|cellTypes, adjust =* *“tukey”))*, which generated pairwise comparisons between genotypes within each cluster (Table S1, DPA analysis tab). Estimates from each GLM for each comparison are pooled using *mice*’s *pool.scalar()* function, and significance for the pooled estimates was calculated using Tukey's test to adjust for multiple comparisons.

### 2.12 Pseudobulk analyses

For pseudobulk analysis DEGs between genotypes are identified using Seurat's FindMarkers function with an FDR cutoff of 0.01, an LFC cutoff of 0.10, and an expression cutoff of 10% of cells per genotype.

### 2.13 Gene ontology network and transcription factor analysis

Overrepresented biological process gene ontology (GO) terms were identified using PANTHER with an FDR cutoff of 0.05.^34^ Genes with significantly increased or decreased expression in h5XFAD, hPS19, or hPS‐5X cells versus hWT controls were compared to the full set of human genes. Identified GO terms were visualized as a network using the Enrichment Map (v3.3.2) plugin for Cytoscape (v3.8.2).^35^ For Transcription factor analysis binding sites within 500 bp upstream of genes differentially expressed in h5XFAD, hPS19, or hPS‐5X cells versus hWT controls were identified using blerr (github.com/englandwe/blerr), using the JASPAR 2018 set of transcription factor binding sites, with a *Z*‐score cutoff of 2.^36^

### 2.14 Bulk RNA‐seq analyses of iMG

RNA was extracted using a standard Trizol extraction protocol and sequenced on an Ilumina Novaseq 6000. Raw bulk RNA‐seq data were initially assessed for quality using FastQC (v0.11.9), and results summarized with MultiQC (v1.10.1). Data then underwent quality trimming and adapter removal using Trimmomatic (v0.39). Adapter sequences were removed by matching them to a reference with a maximum mismatch of two, and the clipping thresholds were set for seed and palindrome to 30 and 10, respectively. Reads were then trimmed using a sliding window of four base pairs, where trimming occurred if the average quality score within the window fell below 30. Bases at the leading and trailing ends of the reads were removed if their quality score was below 15. Finally, reads shorter than 30 bases after trimming were discarded. The trimmed reads were then pseudoaligned to the GRCh38 reference genome with Gencode v29 annotations using Kallisto (v0.48.0) to quantify transcript abundances. Differential expression analysis was performed with DESeq2 (v1.38.3). DEGs were identified for each interferon treatment (alpha, beta, and gamma) versus untreated controls. The top 30 upregulated genes for alpha‐, beta‐, and gamma‐treated samples were selected, and module scores for expression of these three gene sets were calculated in single‐cell RNA‐seq data using Seurat (v4.3). Module scores between the four genotypes were then compared via ANOVA with Tukey post hoc tests. Gene set expression profiles were then projected onto UMAP plots to visualize expression of these genes within the single‐cell dataset. UMAP feature plots were produced using Seurat (v4.3); boxplots were produced using ggplot2 (v3.4.0).

## 3 RESULTS

### 3.1 Generation of xenotolerant mice to examine the interactions between human microglia and AD pathologies in vivo

**FIGURE 1:**
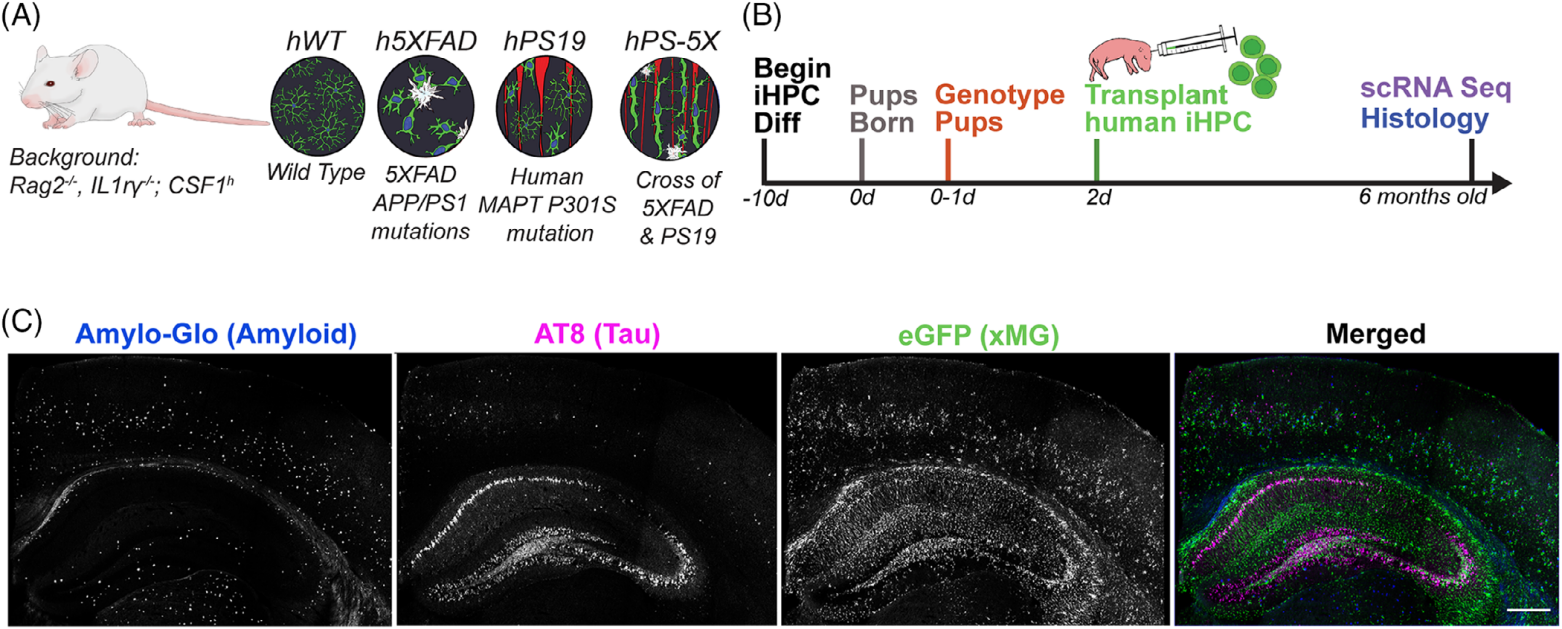
Schematic of the study and experimental design. (A) Schematic of the study. (B) Experimental timeline. (C) Representative confocal stitch within CA1 of the hippocampus of a 6‐month‐old hPS‐5X chimeric mouse, stained for amyloid plaques (Amylo‐Glo), phosphorylated tau (AT8), and xenografted human microglia (xMG) enhanced green fluorescent protein (eGFP). Scale bar = 500 µm

We and others previously demonstrated that iPSC‐derived microglial progenitors can be engrafted into immune‐deficient transgenic mice to examine human‐specific microglial responses to Aβ pathology.^7^, ^37^ However, the interactions between human microglia and neurofibrillary tangles and the combined impact of amyloid and tau pathologies on microglia remain unclear. We therefore sought to develop additional chimeric mouse models to fill this gap. We began by backcrossing the well‐established PS19 model of neurofibrillary tangle pathology^25^ onto a h*CSF1*/Rag2^−/−^/il2rγ^−/−^ (hWT) xenotolerant background to generate “hPS19 mice.” We also used our previously established xenotolerant “h5XFAD mice,” that express co‐integrated mutant transgenes for amyloid precursor protein (APP) and presenilin‐1 (*PSEN1*) on the h*CSF*1/Rag2^−/−^/il2rγ^−/−^ immunodeficient background.^12^, ^38^ We then crossed the resulting hPS19 and h5XFAD models to generate xenotolerant “hPS‐5X mice.” As both PS19 and 5XFAD transgenes are maintained in a hemizygous state and the *hCSF1*, *Rag2*, and *il2r*γ genes are maintained in a homozygous or (‐/y) state, this F1 cross produces four specific genotypes: (1) hWT, (2) h5XFAD, (3) hPS19 mice, and (4) hPS‐5X mice. (Figure 1A).

To examine the potential interactions between human microglia and amyloid, tau, or combined pathologies, mouse pups from each of the four genotypes were transplanted with GFP‐expressing iPSC‐derived microglial progenitors. Importantly, in previous studies we have found no notable changes in hippocampal anatomy or alterations in amyloid load following early postnatal injection of xMG versus saline control.^7^, ^12^, ^38^, ^39^ Following transplantation all mice were aged to 6 months and then tissue was isolated to enable scRNA‐seq of xMG and immunohistochemical (IHC) analyses (Figure 1B). This age was specifically selected to examine the interactions between microglia and AD pathologies, without the potentially confounding immunological effects induced by substantial neuronal death that has previously been reported to reach statistical significance starting at 9 months of age in both 5XFAD and PS19 mice.^25^, ^40^, ^41^ Chimeric mice with combined amyloid and tau pathologies provide a novel model to enable the concurrent investigation of human microglial interactions with both of these hallmark AD pathologies (Figure 1C).

### 3.2 The hPS‐5X chimeric mice exhibit accelerated tau pathology and neuronal loss at 6 months of age

**FIGURE 2:**
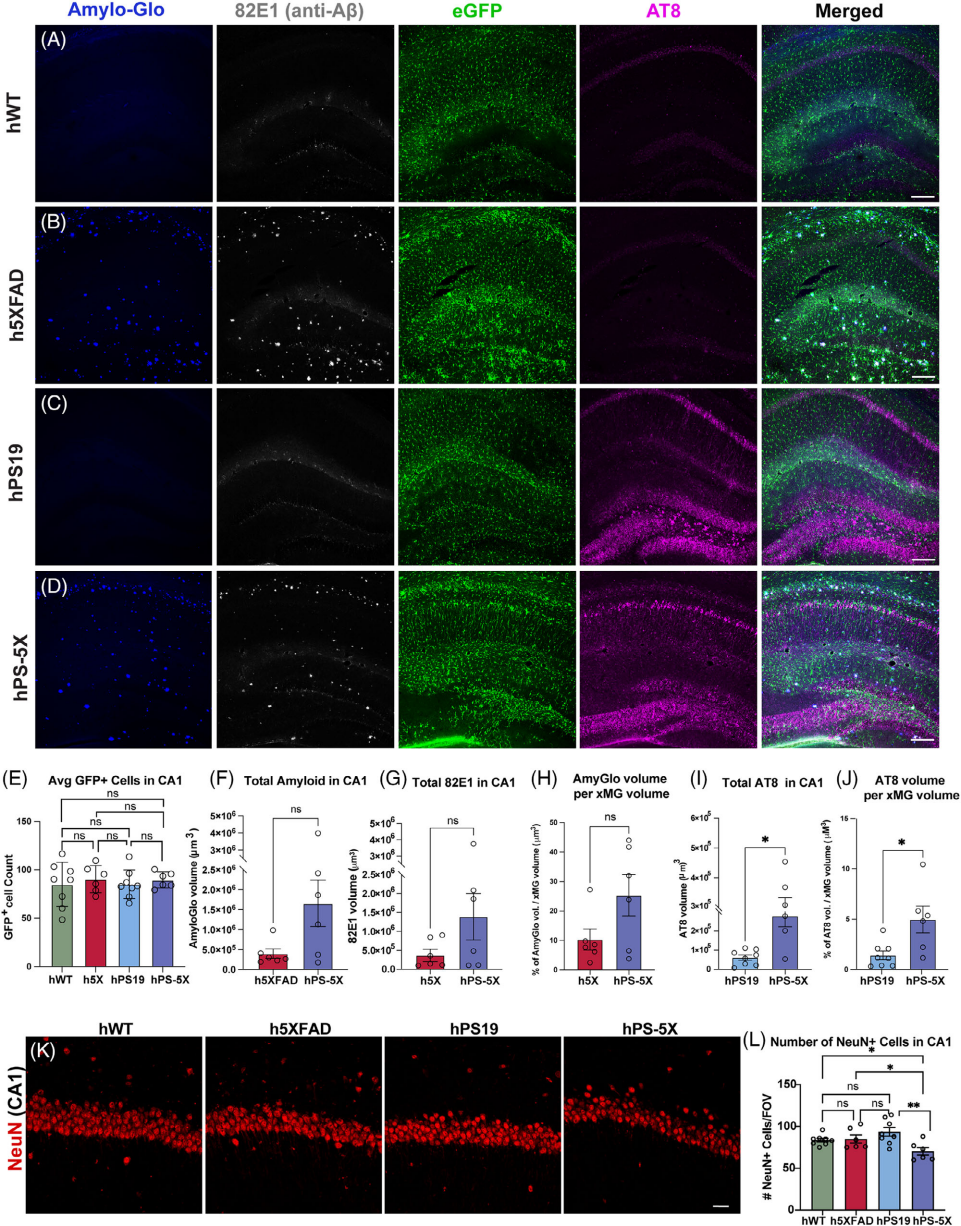
Human microglia interact with amyloid and tau pathologies in 6‐month‐old male mice. (A) Confocal z‐stacks show that xenotransplanted microglia (xenografted human microglia [xMG], green fluorescent protein [GFP]) exhibit a typical tiled distribution in wild‐type mice. (B) In chimeric h5XFAD mice subsets of human microglia are observed adjacent to beta‐amyloid plaques (Amylo‐Glo, blue; 82E1 anti‐Aβ, and white). (C) At 6‐months of age hPS19 mice exhibit sparsely labeled AT8^+^ neurofibrillary tangles (red) within the pyramidal cell layer of CA1. (D) hPS‐5X mice exhibit greater volume of AT8^+^ pathology than hPS19 mice, and xMG surround and interact with both plaques and tangles (Scale bars = 50 µm). (E) There are no significant differences in the total number of engrafted xMG (GFP+ cells) within the four genotypes in the hippocampus (F) Total Amylo‐Glo+ and (G) 82E1^+^ Amyloid volumes remain unchanged between h5XFAD and hPS‐5X mice (*n* = 6). (H) No difference in the total distribution of The Amylo‐Glo volume normalized to xMG volume is detected between h5XFAD and hPS‐5X mice (*n* = 6; *t*‐test, *p* = 0.0833). Both total AT8^+^ tangle volume (I) and AT8 normalized to xMG volume (J) are significantly increased in hPS‐5X hippocampus (*n* = 6) than hPS19 mice (*n* = 8); *t*‐test, *p* = 0.0040 and *p* = 0.0438, respectively. (K) Representative images of NeuN staining in CA1 region of hWT, h5XFAD, hPS19, and hPS‐5X mice (scale bar = 20 µm). (L) A significant decrease in CA1 NeuN^+^ cells were detected in hPS‐5X mice in comparison to hWT, h5XFAD, and hPS19 mice (one‐way analysis of variance [ANOVA], * = *p* ≤ 0.05; ** = *p* ≤ 0.01; *n* = 8, 6, 8, and 6 for hWT, h5XFAD, hPS19, and hPS‐5X genotypes, respectively)

Prior studies have consistently shown that the accumulation of amyloid plaques promotes the development and progression of tau pathology.^21^, ^42^, ^43^, ^44^, ^45^ In contrast, the effects of tau pathology on plaque load appear to vary dependent on the particular mouse models and ages examined.^43^, ^45^, ^46^, ^47^ To determine whether hPS‐5X mice exhibit changes in either of these pathologies, we quantified amyloid plaques, hyperphosphorylated tangles, and GFP‐labeled microglia within the hippocampus and parietal cortex. GFP expression revealed robust human microglial engraftment throughout the forebrain, with equivalent numbers of GFP⁺ microglia detected across all four genotypes within the hippocampus (Figure 2A–E) and cortex (Figure S1 A–E). Quantification of amyloid plaque with either Amylo‐Glo (Figure 2F) or an Aβ‐specific antibody (82E1) (Figure 2G) revealed no significant differences in plaque volume or number between 6‐month‐old h5XFAD and hPS‐5X mice in either the hippocampus (Figure 2A–H) or parietal cortex (Figure S1A–H). However, in both regions, hPS‐5X mice exhibited a non‐significant trend toward increases plaque volume (Figure 2F–H, Figure S1F, G). In contrast to Aβ, and consistent with prior reports, the volume of AT8^+^ hyperphosphorylated tau was significantly increased in both CA1 (Figure 2C, D, I, J) and the parietal cortex (Figure S1C, D, I, J) of hPS‐5X mice in comparison to hPS19 mice.

Next, to determine whether amyloid, tau, or combined pathologies can promote neuronal death by 6‐months of age, the number of NeuN⁺ neurons within CA1 and the parietal cortex were examined across all four genotypes. No significant differences were detected in NeuN density within CA1 between hWT mice with h5XFAD and hPS19 at this timepoint. In contrast, we detected a small, but significant decrease in NeuN^+^ neurons within both CA1 and the parietal cortex of hPS‐5X chimeric mice, suggesting that at 6 months of age, the combination of amyloid and tau pathology promotes neuronal death in hPS‐5X mice (Figure 2K, L; Figure S1K, L).

### 3.3 Amyloid and tau pathologies differentially impact the transcriptional states of human microglia

**FIGURE 3:**
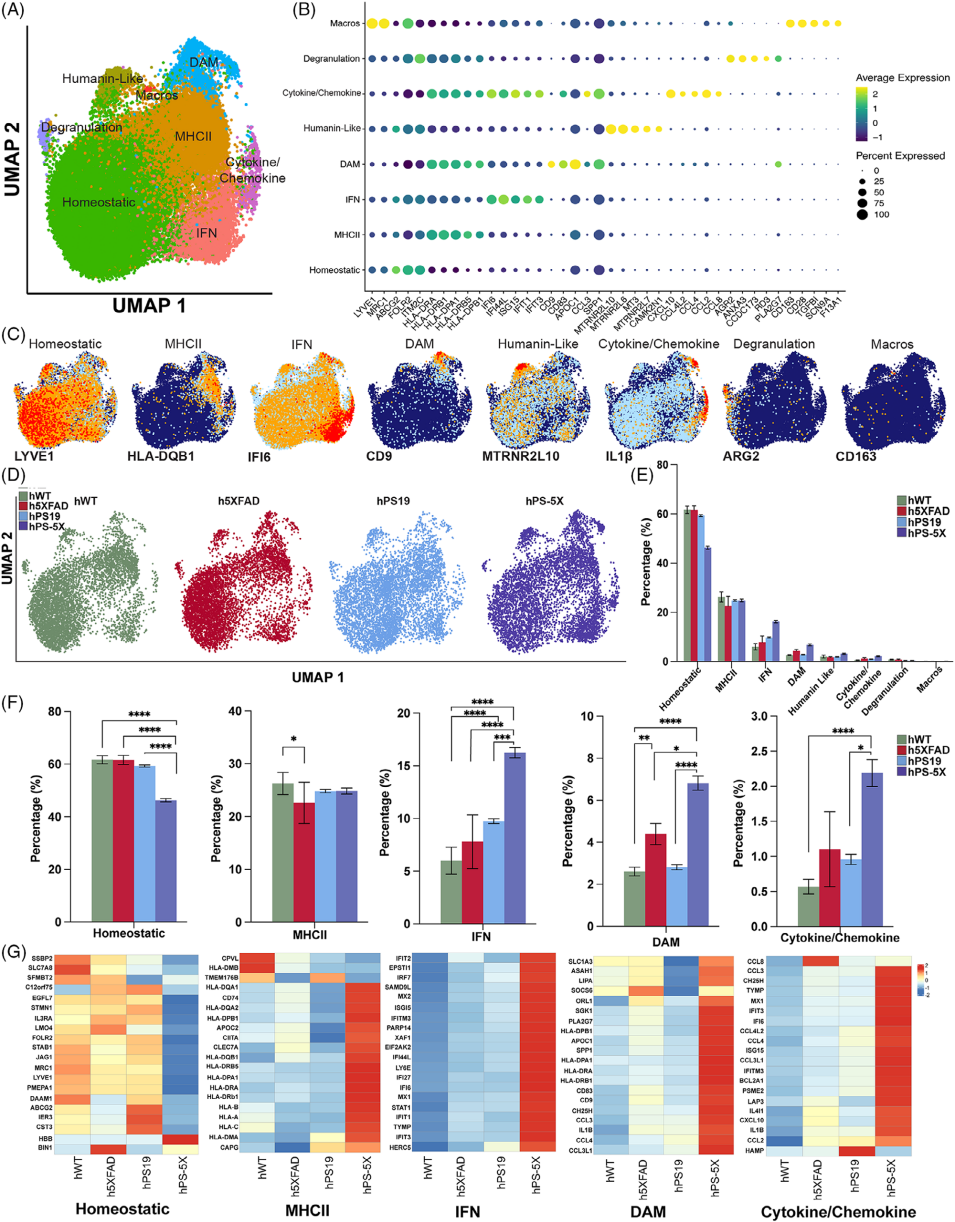
Single‐cell RNA sequencing reveals altered population distributions and gene expression as xenografted human microglia (xMG) respond to combined amyloid and tau pathologies. (A) A uniform manifold approximation and projection (UMAP) plot of 47,863 xMG isolated from 6‐month‐old male hWT, 5X, hPS19, and hPS‐5X chimeric mice reveals eight distinct clusters defined by genes related to MHC class II (MHCII; orange), type I interferon responsive (IFN; Salmon), disease‐associated microglia [DAM] (Blue), humanin‐like (olive green), cytokine/chemokine (Purple), degranulation (Lilac), macros (red), and a “Homeostatic” cluster (green). (B) A dotplot of the top five upregulated differentially expressed genes (DEGs) for each cluster (highest log fold increase for a given cluster versus all other clusters), the size of the dot indicates percent of cells expressing a given gene, color indicates relative expression levels. (C) UMAPs of representative genes for each cluster; *LYVE1* is highly expressed in the homeostatic cluster, *HLA‐DRQ1* is strongly expressed in the MHCII cluster, *IFI6* is expressed highest in the IFN cluster, *CD9* is restricted to the DAM cluster, *MTRNR2L10* is highly expressed in the humanin‐like cluster, *IL‐1β* is expressed highest in the cytokine/chemokine cluster but also within a small subset of DAM, *ARG2* is expressed highest in the degranulation cluster, and *CD163* is expressed highest in the macrophage‐like cluster. (D) Individual UMAPs for each genotype (green; hWT, red; h5XFAD, blue; hPS19, and purple; hPS‐5X) are randomly down‐sampled to show equivalent numbers of cells per genotype. (E) Differential proportion analysis (DPA) was used to perform pairwise comparisons within clusters between each genotype (Table S1). (F) Individual DPA cluster bar plots for Homeostatic, MHCII, IFN, DAM, and cytokine/chemokine are displayed to demonstrate differences more clearly. DPA reveals significantly less homeostatic cells in hPS‐5X mice compared to hWT (*p* = 8.27E‐11), h5XFAD (*p* = 1.18E‐10), and hPS19 mice (*p* = 8.99E‐08). Additionally, h5XFAD mice have less cells in the MHCII cluster (*p* = 0.03256) compared to WT. hPS‐5X mice have significantly more IFN‐responsive human microglia than hWT (*p* = 0), h5XFAD (*p* = 1.09E‐12), and hPS19 mice (*p* = 3.28E‐07) and hPS19 have significantly more IFNs than hWT mice (*p* = 9.45E‐06). hPS‐5X have significantly more DAM than hWT (*p* = 6.34E‐09), h5XFAD (*p* = 0.01044), and hPS19 (*p* = 4.72E‐07), h5XFAD mice additionally have more DAM than hWT mice (*p* = 0.00236). Finally, hPS‐5X mice have significantly more cytokine/chemokines than hWT (*p* = 5.75E‐05) and hPS19 (*p* = 0.04481). (G) Heatmaps comparing relative gene expression of the top 20 DEGs (based on LogFC) in homeostatic, MHCII, IFN, DAM, and cytokine/chemokine clusters between hWT, h5XFAD, PS19‐hCSF1, and hPS‐5X mice reveal a robust induction of many activation genes in hPS‐5X mice. * = *p* ≤ 0.05; ** = *p* ≤ 0.01; **** = *p* ≤ 0.0001. Error bars in E,F = standard deviation

To characterize the response of human xMG to amyloid, tau, or combined pathologies we isolated xMG from male, 6‐month‐old hWT, h5XFAD, hPS19, and hPS‐5X chimeric mice and performed scRNA‐seq on the isolated cells. Following quality control filtering, we identified 12,422 hWT, 11,045 h5XFAD, 18,599 hPS19, and 5797 hPS‐5X microglia with average gene per cell counts of 1661, 1658, 1137, and 1512, respectively (Table S1). A uniform manifold approximation and projection (UMAP) plot of the combined 47,863 xMG isolated from all four genotypes reveals multiple clusters of human microglia (Figure 3A) that are enriched in genes related to MHC class II, type I interferon responses, DAM, humanin‐like, cytokine/chemokine, degranulation (genes enriched during neutrophil degranulation), and border associated macrophages. Consistent with prior studies,^12^, ^38^ homeostatic microglia made up the most abundant cluster and were defined by canonical homeostatic markers such as *OLFML3*, *VSIG4*, and *FOLR2* and very low levels of microglial activation genes (Figure 3A–E, Table S1). The top 5 DEGs for each cluster were determined by the greatest average log‐fold change for that cluster versus all other clusters (Figure 3B). While many of these cluster designations have previously been described in the literature, the small “humanin‐like” and “degranulation” clusters were named based on the DEGs enriched within these clusters relative to all other clusters. For example, several humanin genes, which are associated with antioxidant and pro‐resolving functions in efferocytotic macrophages, exhibited enriched expression within the humanin‐like cluster.^48^ The degranulation cluster designation likewise was chosen based on the enrichment of DEGs that have previously been implicated in neutrophil degranulation, a process in which neutrophils release proteases, cytokines, and chemokines into the extracellular space.^49^ Both Dot plot (Figure 3B) and gene‐specific UMAP (Figure 3C) analyses reveal additional heterogeneity and some overlap of individual genes between clusters. For example, *IL‐1β* expression is most highly enriched in the cytokine/chemokine cluster but also elevated within a subset of DAM microglia.

To visualize potential differences in cluster distributions between genotypes, UMAPs were randomly down sampled to equivalent cell numbers per group (Figure 3D). We then utilized DPA to perform pairwise comparisons between genotypes for each cluster.^12^, ^38^ DPA revealed a significant decrease in homeostatic microglia within hPS‐5X mice in comparison to hWT, h5XFAD, and hPS19 mice (Figure 3E–F). Additionally, xMG from h5XFAD mice exhibited fewer cells within the MHCII cluster in comparison to hWT mice. hPS‐5X mice also exhibit significantly more IFN‐enriched xMG than hWT, h5XFAD, and hPS19 mice. Interestingly, at this age tau pathology alone had no effect on the number of microglia expressing DAM genes but led to significantly heightened expression of IFN‐responsive transcripts in comparison to hWT mice (Figure 3F). Thus, at 6 months of age the DAM phenotype is predominantly associated with amyloid pathology, whereas IFN genes are predominantly induced by tau pathology. However, hPS‐5X mice also exhibited significantly more DAM and INF‐responsive microglia than all other groups (Figure 3F), suggesting that the combination of amyloid and tau pathologies synergize to promote the induction of both of these microglial responses. As expected and consistent with our prior studies,^7^ h5XFAD mice also exhibited increased numbers of DAM microglia than hWT mice. Interestingly, hPS‐5X mice also exhibited a significantly increased induction of cytokine/chemokine enriched microglia in comparison to both hWT and hPS19 mice (Figure 3F). Next, heatmaps were generated to compare the expression of the top 20 DEGs within the Homeostatic, MHCII, IFN, DAM, and cytokine/chemokine clusters compared to every other cluster (Figure 3G, Table S1). As expected, hPS‐5X engrafted microglia exhibited a broad downregulation of many of the genes that define the Homeostatic cluster in our dataset. Conversely, hPS‐5X engrafted microglia, showed a robust increase in the expression of MHC class I and II genes in comparison to hWT, h5XFAD, and hPS19 groups. While PS19 mice exhibit increased expression of multiple IFN responsive transcripts including *MX1, MX2, IRF7*, and *STAT1* relative to hWT mice, each of these genes is further elevated in hPS‐5X mice (Figure 3G, Table S1). Consistent with our prior studies, many DAM‐related genes are upregulated in h5XFAD engrafted microglia in comparison to hWT mice. In contrast, only a small subset of these genes were elevated in 6‐month‐old hPS19 mice. As with the interferon‐responsive genes, DAM‐related transcripts are even further elevated in hPS5X mice, with 19 out of the 20 DAM‐related genes exhibiting increased expression in hPS5X derived microglia in comparison to h5XFAD derived microglia (Figure 3G, Table S1).

### 3.4 Pseudobulk analysis reveals both similarities and differences in the microglial response to amyloid and tau pathologies

**FIGURE 4:**
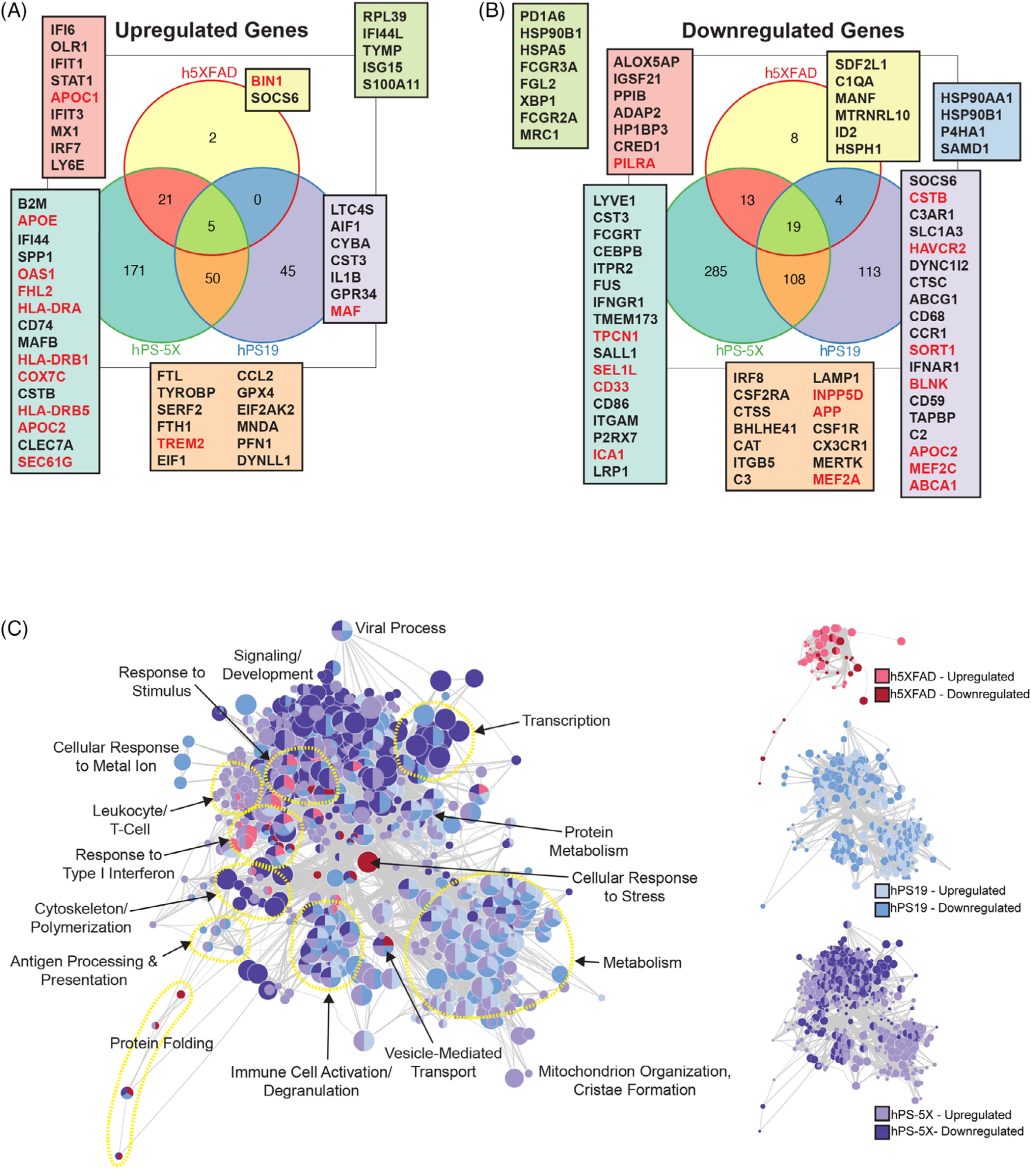
Pseudobulk analysis reveals both similarities and differences in the microglial response to amyloid and tau pathologies. (A) Overlapping upregulated and (B) downregulated xenografted human microglia (xMG) genes of each genotype compared to hWT, genes of interest are highlighted in boxes in corresponding colors, AD risk genes are inscribed in red. (C) Gene ontology (GO) network analysis generated from all pseudobulk differentially expressed genes (DEGs), nodes represent individual GO terms, whereas the size represents the number of genes within a node, and the distance between nodes represents similarity, similar nodes are encircled with yellow dashed lines labeled with arrows

To further understand whether human microglia exhibit differing responses to amyloid, tau pathology, or the combination of both of these hallmark AD pathologies, we next performed pseudobulk analysis of our scRNA‐seq datasets. Numerous DEGs are identified when comparing each individual genotype to hWT controls (Table S2). Specifically, we detected 72 DEGs in h5XFAD mice in response to amyloid pathology alone, 344 significantly altered genes in hPS19 mice, and 672 DEGs when microglia interact with both AD pathologies in hPS‐5X mice. Interestingly, Venn diagram comparisons of upregulated and downregulated genes across each AD genotype revealed varying degrees of overlap and a robust enrichment of AD risk genes (labeled in red) (Figure 4A and B).^50^ Upregulation of *OAS1* in the hPS‐5X mice is particularly noteworthy, as previous reports have linked *OAS1* variants with AD risk and *OAS1* is a key antiviral factor induced by interferons that can sense exogenous nucleic acids.^51^, ^52^ Extracellular nucleotides have also previously been implicated in type I IFN responses to amyloid and tau pathology in both mice and humans.^11^, ^23^, ^53^, ^54^, ^55^

To better understand the functional implications of these transcriptional changes, we next performed GO network analysis (Figure 4C; Table S2). The xMG within h5XFAD mice are predominantly characterized by an upregulation of pathways such as “response to stimulus” and “response to type 1 interferon” while downregulating genes related to “cellular response to stress” and “protein folding.” The “response to type 1 interferon” in h5XFAD mice is also shared by hPS19 mice but is further expanded in hPS‐5X mice. hPS19 and hPS‐5X engrafted microglia also share alterations in “metabolism” and in hPS‐5X mice “mitochondrion organization, and cristae formation” are upregulated. Additional nodes under “metabolism” include GO terms surrounding RNA and DNA catabolism, which may explain the overall skew towards downregulated genes in both hPS19 and hPS‐5X xMG. Indeed, interactions between pathogenic tau and RNA binding proteins that lead to diminished gene expression have been described by multiple groups.^42^, ^43^, ^44^, ^56^

### 3.5 Microglia can adopt a unique rod‐like morphology in hPS‐5X chimeric mice

**FIGURE 5:**
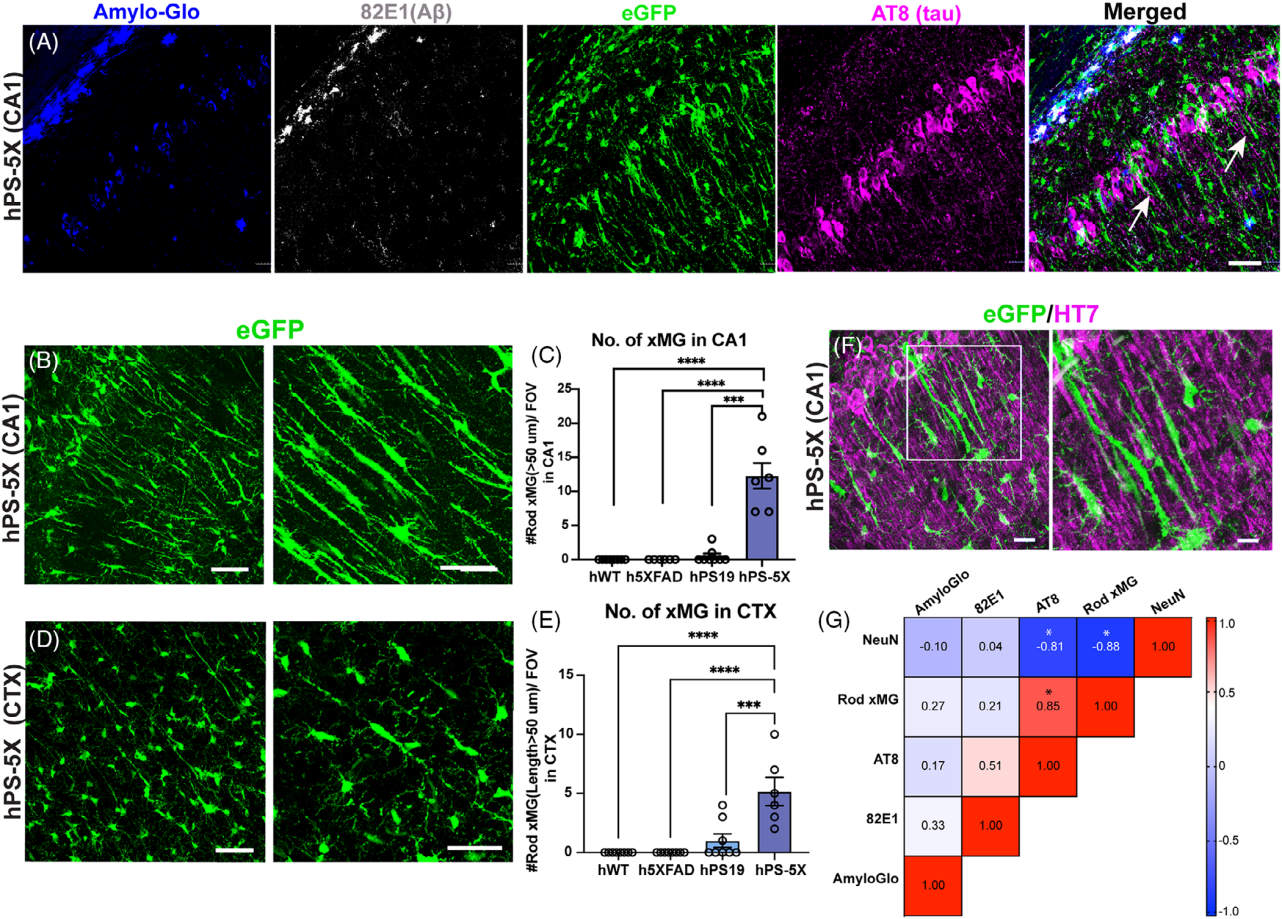
Human microglia adjacent to tau^+^ neuronal dendrites in hPS‐5X mice exhibit rod shaped morphologies. (A) Green fluorescent protein (GFP) ‐expressing xenografted human microglia (xMG) (green) adopt rod‐like morphologies (white arrows) within CA1 of 6‐month‐old hPS‐5X mice. (B) High magnification representative images of rod‐like xMG within the stratum radiatum of CA1 of hPS‐5X mice. (C) Quantification of rod xMG with length > 50 µm in CA1 of all genotypes (*n* = 8, 6, 8, and 6 for hWT, h5XFAD, hPS19, and hPS‐5Xgenotypes, respectively). (D) High magnification representative images of rod‐like xMG in the cortex of hPS‐5X mice. (E) Quantification of rod xMG with length > 50 µm in the cortex of all genotypes (*n* = 8, 6, 8, and 6 for hWT, h5XFAD, hPS19, and hPS‐5X genotypes, respectively; one‐way analysis of variance (ANOVA); *** = *p* ≤0.001, **** = *p* ≤ 0.0001). (F) Rod‐like xMG are frequently observed adjacent to the proximal dendrites of tau^+^ pyramidal neurons (GFP, green; tau‐HT7, magenta). (G) Matrix of Pearson correlation coefficients of quantified Amylo‐Glo, 82E1, AT8, NeuN, and rod xMG, of hWT, h5XFAD, hPS19, and hPS‐5X mice (*n* = 8. 6, 8, and 6 for hWT, h5XFAD, hPS19, and hPS‐5Xgenotypes respectively; *p*‐value cutoff for significance = 0.05). Scale bar = 50 µm

Confocal imaging of GFP‐expressing human microglia within hPS‐5X mice revealed a highly distinct elongated or “rod‐like” morphology that was especially prevalent within the stratum radiatum of CA1, but also present in the cortex (Figure 5A–D). This characteristic microglial shape has previously been described in varying pathological states, including AD, but the functional implications of rod microglia remain mostly unclear.^57^, ^58^, ^59^, ^60^ To determine the relative number of rod microglia across each genotype we utilized IMARIS software to quantify the number of microglia that exhibited a maximal process length over 50 µm. This specific cutoff was selected based on the average length of the xMG calculated via IMARIS image analysis software (Figure S2). While human microglia rarely exhibited such elongated processes in hWT, h5XFAD, and hPS19 mice, microglia with lengths of 50 µm or greater were frequently detected in hPS‐5X mice (Figure 5B, C, Figure S2). Although less abundant, rod microglia were also observed within the parietal cortex of hPS‐5X mice but rarely detected in the other groups (Figure 5D, E).

The somatodendritic accumulation of hyperphosphorylated tau within the neuronal perikarya of CA1 pyramidal neurons and their proximal dendrites within the stratum radiatum has been well described in hPS19 mice and many other tauopathy models.^61^, ^62^ As rod microglia were also abundant within this region we wondered whether this might indicate a potential relationship between tau pathology and rod morphology. Indeed, co‐labeling of GFP^+^ microglia and human tau (HT7) revealed a close juxtaposition between rod microglia and tau immunoreactive dendrites within the stratum radiatum of hPS‐5X mice (Figure 5F). We therefore compared the number of rod microglia to the volume of AT8 immunoreactive tangles, the density of CA1 neurons, and the prevalence of amyloid pathology across all mice. While measures of amyloid pathology showed no significant relationship with rod microglia numbers, we discovered a highly significant positive correlation between the number of xMG with rod morphology and the density of AT8^+^ tangles in CA1 (*r* = 0.85, *p* = 0.03) (Figure 5G). Interestingly, neuronal density within CA1 was also inversely correlated with the number of rod microglia (*r* = –0.88, *p* = 0.02), suggesting that rod microglia may form in response to signals from degenerating tau‐accumulating dendrites. Consistent with this notion, neuronal density within CA1 was also inversely correlated with AT8 levels (*r* = –0.81, *p* = 0.05).

### 3.6 Rod microglia upregulate interferon‐responsive proteins

**FIGURE 6:**
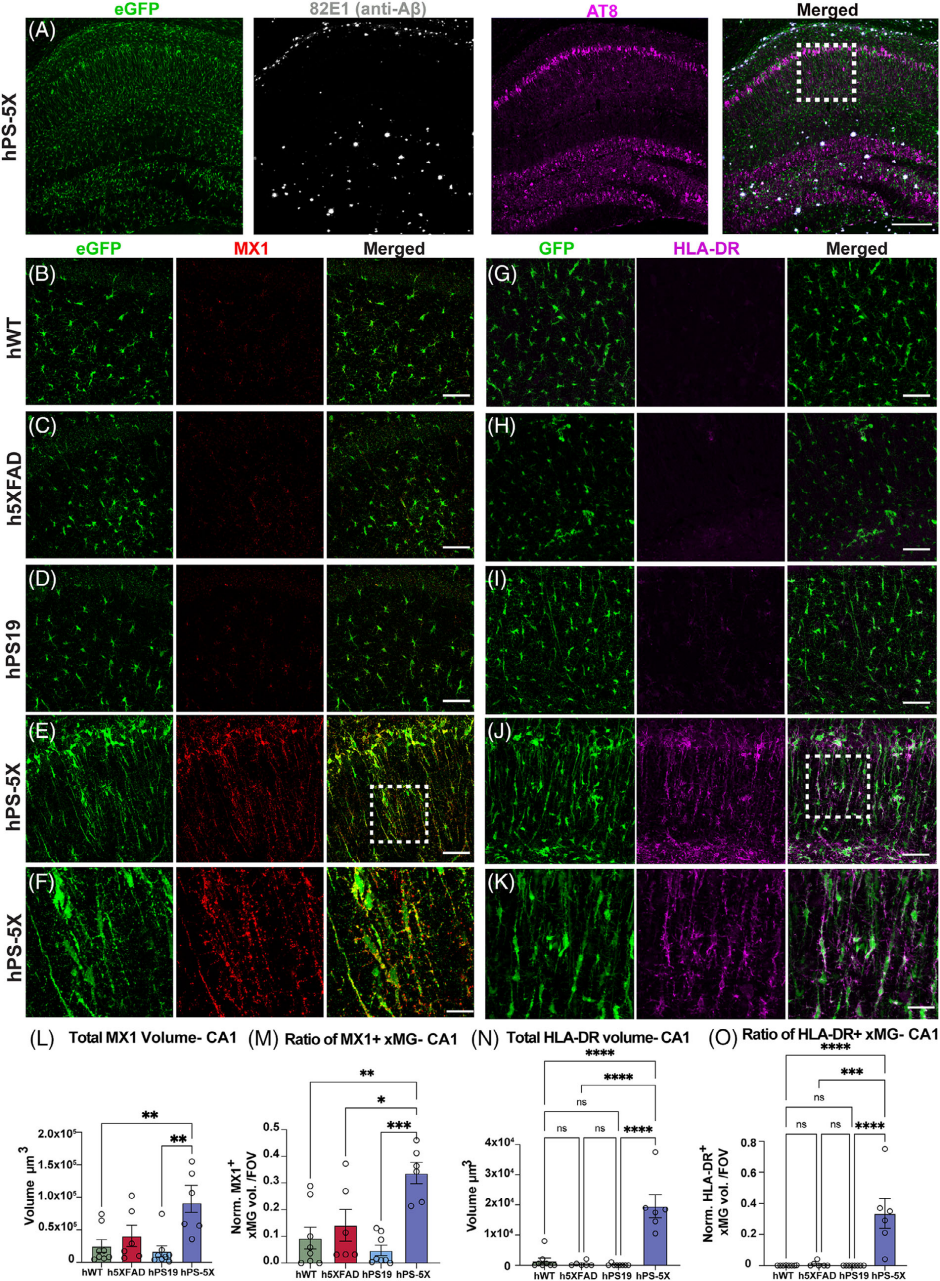
Rod microglia express interferon (IFN) responsive proteins. (A) The region within CA1 wherein the MX1 and HLA‐DR images were collected is shown with dashed lines. (B) Xenografted human microglia (xMG) (green, enhanced green fluorescent protein [eGFP]) within CA1 of hWT mice, (C) h5XFAD mice, and (D) hPS19 mice exhibit expression of MX1 (red). (E) xMG in hPS‐5X mice, exhibit a substantial increase in MX1 immunoreactivity. (F) Representative snapshot with higher magnification (2.5× zoom) show the MX1 immunostaining (red) overlaps with rod‐like microglia (eGFP, green). Scale bars = 50 µm. (G) xMG (green, eGFP) within CA1 of hWT mice, (H) h5XFAD mice, and (I) hPS19 mice exhibit expression of HLA_DR (magenta). (J) xMG in hPS‐5X mice, exhibit a substantial increase in HLA‐DR immunoreactivity. (K) Representative snapshot with higher magnification (2.5× zoom) show the HLA‐DR immunostaining (magenta) overlaps with rod‐like microglia (eGFP; green). Scale bars = 50 µm. (L) Average MX1 volume in CA1 is significantly higher in the hPS‐5X mice compared to h5XFAD, hPS19, and WT. (M) Ratio of MX^+^ xMG is also significantly higher in hPS‐5X mice compared to hWT, h5XFAD, and hPS19 mice. (N) Quantification of total HLA‐DR volume within CA1 show significantly higher levels in hPS‐5X mice compared to all other genotypes (*n* = 8, 6, 8, and 6 for hWT, h5XFAD, hPS19, and hPS‐5X genotypes, respectively; one‐way ANOVA, * = *p* ≤ 0.05; ** = *p* ≤ 0.01; *** = *p* ≤ 0.001). (O) Ratio of HLA‐DR^+^ xMG is also significantly higher in hPS‐5X mice compared to hWT, h5XFAD, and PS19‐hCSF1

Our scRNA‐seq analyses revealed significant increases in several IFN‐responsive transcripts including *MX1*, a type I IFN responsive gene implicated in antiviral responses and upregulated in AD microglia,^63^ as well as *HLA‐DR*, a class II major histocompatibility complex and AD risk gene that is also commonly induced by interferon signaling.^64^ We therefore sought to determine whether these transcripts were also increased at the protein level within rod microglia. IHC analyses of CA1 (boxed region in Figure 6A), where rod microglial morphology was most abundant, revealed very low levels of MX1 immunoreactivity in hWT, 5X, and hPS19 engrafted microglia (Figure 6B–E). In contrast, rod microglia within hPS‐5X mice exhibited significantly increased expression of MX1 (Figure 6E, F, L, M). We and others have previously shown that HLA‐DR expression is upregulated in plaque‐adjacent xMG.^7^, ^37^ While a similar upregulation of HLA‐DR in response to amyloid plaques was observed in other brain regions (data not shown), very few plaques are observed within CA1, and thus little HLA‐DR expression was detected in this region of 5X mice (Figure 6G). HLA‐DR was similarly absent within CA1 of hWT and hPS19 mice but greatly increased in hPS‐5X engrafted rod microglia (Figure 6H–K, N, O).

### 3.7 Rod morphology can be induced in vitro by treatment of human microglia with type I but not type II IFNs

**FIGURE 7:**
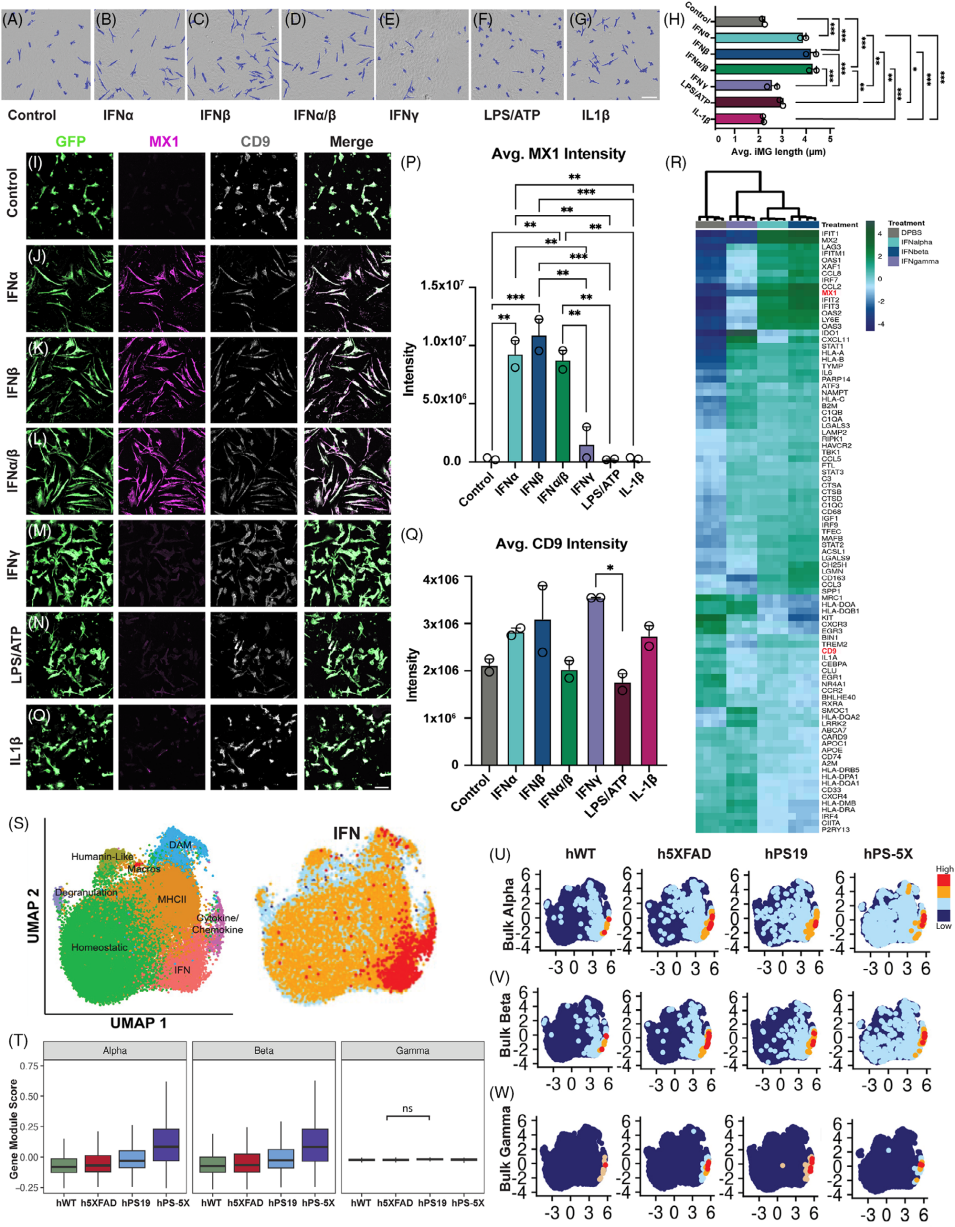
Type 1 interferons (IFNs) induce rod microglia in vitro. Representative phase contrast mask of iMG in vitro treated with (A) phosphate buffered saline (PBS), (B) IFN alpha (IFNα), (C) IFNβ, (D) IFNα/β, (E) IFNγ, (F) lipopolysaccharide/adenosine triphosphate (LPS/ATP), or (G) interleukin (IL)‐1β. (H) Treatment with type 1 IFNs leads to a significant increase in length to width ratios compared to all other conditions (*n* = 2 wells per group, fouor images averaged per well. Scale A–G = 100 µm. * = *p* ≤ 0.05; ** = *p* ≤ 0.01; *** = *p* ≤ 0.001; **** = *p* ≤ 0.0001. (I–O) Confocal microscopy of green fluorescent protein (GFP), MX1, and CD9 in iMG (*n* = 2 wells per group, four images averaged per well. Scale I‐O = 20 µm. * = *p* ≤ 0.05; ** = *p* ≤ 0.01; *** = *p* ≤ 0.001; **** = *p* ≤ 0.0001). (P) Quantification of MX1 (IFNα vs. Control *p* = 0.0023; IFNα vs. IFNγ *p* = 0.0064; IFNα vs. LPS/ATP *p* = 0.0026; IFNα vs. IL‐1β *p* = 0.0023, IFNβ vs. Control *p* = 0.0008; IFNβ vs. IFNγ *p* = 0.0021; IFNβ vs. LPS/ATP *p* = 0.0009; IFNβ vs. IL‐1β, *p* = 0.0008, IFNα/β vs. Control *p* = 0.0032; IFNα/β vs. IFNγ *p* = 0.0094; IFNα/β vs. LPS/ATP *p* = 0.0037; IFNα/β vs. IL‐1β *p* = 0.0033; *F*[6, 7] = 28.45). (Q) Quantification of CD9 (*p* = 0.0378; *F*[6, 7] = 4.571). (R) Heatmap of a subset of DEGs from RNA‐seq analyses of IFNα, IFNβ, or IFNγ treated iMG. (S) Uniform manifold approximation and projection (UMAP) obtained from scRNA‐seq of xenografted human microglia (xMG) extracted from all genotypes. The IFN responsive genes subcluster distribution is presented on the UMAP. (T) The bulk RNA‐seq data from IFN treated iMG was used to calculate Gene module scores and genotypes compared by analysis of analysis of variance (ANOVA) with Tukey post hoc tests (*p* < 0.0001 for all pairwise comparisons except those labeled ns). (U) Integrated bulk RNA‐seq data from IFNα, (V) IFNβ, and (W) IFNγ treated cultured iMG were divided into four equal bins, each represented by a color (blue, lowest expression;red, highest expression). These scores were then plotted onto the scSeq UMAPs for each genotype revealing a robust induction of IFNα/β responsive transcripts in hPS‐5Xengrafted xMG

Given the high expression of interferon‐responsive proteins within rod microglia and the expanded IFN‐responsive xMG cluster in hPS‐5X mice, we wondered whether a similar rod‐like phenotype could be elicited in vitro by exposure to disease‐relevant stimuli. We therefore treated cultured human iMG for 48 h with various stimuli (Figure 7). Control treated iMG maintained a variety of differing morphologies, reflecting the typical appearance of cultured microglia (Figure 7A). In contrast, treatment with 100 ng/mL of the type I IFNs IFNα2A (Figure 7B), IFNβ (Figure 7C), or a combination of IFNα and IFNβ (Figure 7D), led to adoption of a striking rod‐like morphology in vitro. Interestingly, this elongation was not driven by type II IFN, as 100 ng/mL of IFNγ did not elicit a rod‐like morphology (Figure 7E). Likewise, neither induction of the inflammasome via a classical paradigm (LPS + ATP), nor direct treatment with IL‐1β, induced rod‐liin vitroke morphology (Figure 7F–G).^65^, ^66^ To quantify the effect of each treatment on morphology the longest axis of each microglia was measured and averages for each well and each treatment group were calculated. This analysis further demonstrated that treatment with IFNα and/or IFNβ produced significantly more elongated microglia than control treatments (Figure 7H). In contrast, treatment with IFNγ, LPS/ATP, or IL‐1β produced no significant changes in average microglial length (Figure 7H). Given the high level of *MX1* expression observed in rod‐xMG in vivo, we next examined the expression of this type 1 IFN‐responsive protein along with the DAM marker CD9, via confocal microscopy (Figure 7I–O). MX1 protein expression was almost undetectable in control‐treated cells whereas treatment with IFNα, IFNβ, or IFNα/β greatly increased MX1 immunoreactivity while eliciting little to no change in CD9 expression. In contrast, treatment with IFNγ, LPS/ATP, or IL‐1β had little effect on either MX1 or CD9 expression (Figure 7P–Q). To further understand the impact of IFN treatments on human microglia, we next performed bulk RNA‐seq analysis of control, IFNα, IFNβ, or IFNγ treated iMG. The resulting DEGs revealed clear differences in the transcriptional response of iMG to type I versus type II interferons (Figure 7R, Table S3). For example, IFNα and IFNβ treatment led to a far greater induction of *MX1, OAS1/2/3, CCL2/3, CD163*, and *SPP1* than IFNγ treatment (Figure 7R). In contrast, all three treatments led to an equivalent upregulation of many lysosomal genes including *LAMP2, CTSA, CTSB, CTSD*, and *CD68*. To further compare these bulk RNA‐seq data to our in vivo xMG analysis, module scores were generated to capture the effect of IFN treatment and then integrated and plotted onto our scRNA Seq analysis for each genotype (Figure 7S–W). This integration revealed a greater percentage of xMG that exhibit gene signatures induced by IFNα or IFNβ treatment than IFNγ treatment and further revealed a progressive increase in type I interferon responsive module scores from hWT engrafted mice to h5XAD mice, followed by hPS19 mice and culminating in the greatest IFN‐responsive module enrichment in hPS‐5X mice (Figure 7T–W).

## 4 DISCUSSION

Genetic studies continue to highlight the importance of microglia in AD, motivating a growing investigation and understanding of murine microglial responses to amyloid or tau pathologies.^7^, ^16^, ^19^, ^21^, ^22^ However, many of these GWAS risk genes exhibit limited homology with their murine counterparts.^13^ Single nuclei RNA sequencing studies of *post mortem* human samples have likewise shown relatively limited overlap in microglial transcriptomic profiles between mouse models and human disease.^67^, ^68^, ^69^ We previously developed a chimeric approach to examine the impact of Aβ plaque pathology on human iPSC‐derived microglia in vivo,^21^ yet far less is known regarding how human microglia respond to neurofibrillary tangles or the combination of these two hallmark AD pathologies. In the current study, we sought to address this gap by developing and examining new chimeric models of tau pathology and combined amyloid and tau pathologies.

Here we show that amyloid and tau pathologies individually induce a differential transcriptomic response in human microglia. Specifically, we find that at 6 months of age, prior to onset of neuronal death in either of the h5XFAD or hPS19 mice, amyloid pathology induces both DAM and interferon‐responsive states, whereas tau pathology alone induces interferon response programs without eliciting a DAM‐like phenotype. More importantly, when amyloid and tau pathologies are combined, mimicking human AD, microglia exhibit numerous changes in transcriptional state including the further induction of Interferon Responsive programs, significant expansion of DAM microglia, and stimulation of a proinflammatory cytokine/chemokine‐enriched phenotype. Pathological assessment showed increased amyloid‐driven tau accumulation in the hPS‐5X mice consistent with prior reports,^43^, ^70^, ^71^ as well as a significant increase in neuronal loss in hPS‐5X mice compared to all other genotypes. In addition, the combination of amyloid and tau pathologies leads to a significant increase in rod microglial morphology within the hippocampus. Confocal microscopy further demonstrates the intimate interactions between rod microglia and tau‐laden neurofibrillary tangles, suggesting that rod microglia likely impact neuronal function.

Rod microglia have previously been described in varying pathological states, including AD, Huntington's disease, TDP‐43 neurodegeneration, and traumatic brain injury,^58^, ^59^, ^60^ In the current study we further demonstrate that rod morphology is strongly correlated with both AT8 pathology and hippocampal neurodegeneration. In contrast, the development of rod microglia is not correlated with amyloid plaque load. The rod microglia in hPS‐5X also express a variety of activation signals including interferon responsive proteins and MHCII antigen presentation molecules. Importantly, rod morphology can also be induced in human iMG by type I interferons, but not other inflammatory stimuli, providing initial mechanistic insight into the origin of this distinct microglial morphology. However, whether or not amyloid, tau or combined pathologies can directly induce rod iMG morphology remains unclear. Type I but not type II IFN‐treated iMG also exhibit DEG patterns that closely resemble the transcriptional changes observed in xenotransplanted human microglia isolated from hPS‐5X mice in comparison to all other genotypes. Despite these intriguing new findings, additional research will be needed to fully understand the functional implications of rod microglia. For example, one prior study suggested that rod microglia may promote neuronal survival, albeit at the expense of reduced synapse density,^72^ Alternatively, rod microglia may instead promote tangle pathology by producing proinflammatory cytokines that induce tau hyperphosphorylation.^73^

An important limitation of the current study is the relatively low sample size utilized in our scRNA‐seq analysis. However, our IHC validation of key transcriptional findings with larger group sizes greatly increases confidence in our key findings. Despite robust and consistent engraftment across all groups, the potential impact of remaining murine microglia on human microglial responses to amyloid and tau pathologies also remains unclear. Future studies that take advantage of new approaches that enable the complete replacement of murine microglia with human xMG^73^, ^74^ will likely help to address this remaining question. Taken together, the development of these new chimeric models along with our transcriptional, histological, and morphological assessments provide novel insights into the transcriptional and functional changes that occur in human microglia in response to AD pathologies. We also anticipate that these models will provide promising new in vivo platforms to further define and manipulate the interactions between human microglia, AD risk genes, and plaque and tangle pathologies.

## CONFLICT OF INTEREST STATEMENT

Mathew Blurton‐Jones is a co‐inventor of patent WO/2018/160496, related to the differentiation of human pluripotent stem cells into microglia. Mathew Blurton‐Jones and Robert C. Spitale are co‐founders of Savanna Biotherapeutics. The authors have no additional financial interests. Author disclosures are available in the Supporting Information.

## ETHICS APPROVAL AND CONSENT TO PARTICIPATE

All animal procedures were conducted in accordance with the guidelines set forth by the National Institutes of Health and the University of California, Irvine Institutional Animal Care and Use Committee, who approved the study protocol. The GFP‐expressing iPSC line was purchased from Coriell (AICS‐0036 GFP line) and the use of pluripotent stem cells for these studies was approved by UC Irvine's human Stem Cell Research Oversight (hSCRO) committee.

## CONSENT FOR PUBLICATION

All authors have approved the contents of this manuscript and provided consent for publication.

## Supporting information



Supporting Information

Supporting Information

Supporting Information

Supporting Information

Supporting Information

## Data Availability

The RNA‐seq data have been deposited in Gene Expression Omnibus (GEO). The code for data analysis has been uploaded to GitHub. All other data associated with this study are presented in the main text or Supplementary Materials. Requests for materials will be fulfilled by the corresponding author with appropriate material transfer agreements.
