# Supplementary material for: Human microglia differentially respond to β‐amyloid, tau, and combined Alzheimer's disease pathologies in vivo
Source: Alzheimers Dement. 2025 Nov 21;21(11):e70930. doi: 10.1002/alz.70930 (PMC12635866; doi:10.1002/alz.70930)
Supplement: Supplementary file 5 — Supporting Information [file ALZ-21-e70930-s001.pdf]

Supplementary Figure 1.

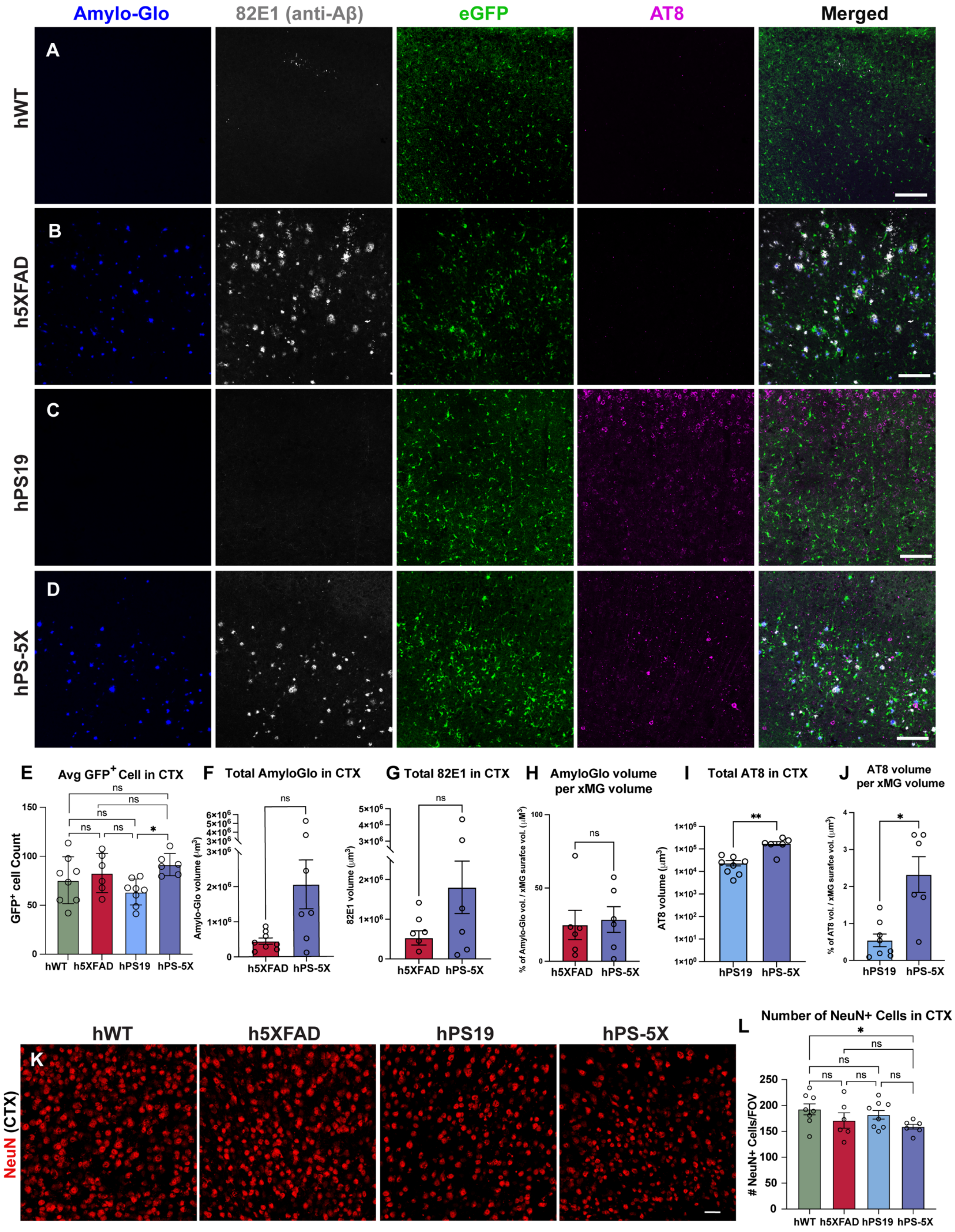

Suppl. Fig. S1. Human microglia interact with both amyloid and tau pathology within the cortex. **(A)** Representative confocal image captured from the Posterior Parietal Association Area of the cortex (PP CTX) overlying the dorsal hippocampus of 6-month-old chimeric mice. Human GFP-expressing microglia (green**)** are seen uniformly tiling the hWT murine cortex. **(B)** In h5XFAD mice, xMG are observed near amyloid beta plaques (Amylo-Glo in blue and 82E1 in white). **(C)** xMG in the hPS19 cortex show presence of a few rod-like morphology xMG and AT8 pathology (red). **(D)** The AT8 pathology (red) are more profound and developed in the cortex of hPS-5X mice. The xMG approach and surround amyloid beta plaques in the hPS-5X cortex but have minimal interaction with tangles. **(E)** There is a small but significant increase in xMG numbers within the cortex of hPS-5X mice in comparison to hPS19 mice. This difference seems to be driven by a slight increase in the hPS-5X mice that is not significantly different from hWT or h5XFAD groups in combination with a slight decrease of xMG in the cortex of hPS19 mice which is also not significantly different from hWT and h5XFAD mice **(F)** No significant differences observed in total amyloid levels within the cortex between h5XFAD and hPS-5X mice (n=6; t-test; p = 0.598). **(G)** Total Amyloid beta levels (82E1) remain unchanged between h5XFAD and hPS-5X mice (n=6; t-test; p = 0.1065). **(H)** No difference in the total distribution of xMG normalized to Amylo-Glo volume is detected between h5XFAD and hPS-5X mice (n=6 and 6 respectively; t-test, p = 0.7867). **(I)** Total AT8 immunoreactive volume is significantly increased in hPS-5X mice compared to hPS19 mice (n=6 and n=8 respectively; t-test; p= 0.0030). **(J)** The xMG volume normalized to AT8 pathology shows significant increase in the hPS-5X mice compared to hPS19 (n=6 and 8 respectively; t-test, p = 0.0124). **(K)** Representative images of NeuN staining in posterior parietal association areas (layer 6) of cortex directly vertical to the

imaged CA1 region above the corpus callosum, in hWT, h5XFAD, hPS19and hPS-5X mice. Scale bar= 20 µm). **(L)** No differences in NeuN+ cell numbers were detected between wild-type, h5XFAD, or PS1 mice. In contrast, a significant decrease in cortical NeuN+ cells was detected in hPS-5X mice in comparison to hWT controls (n=8, 6, 8 and 6 for hWT, h5XFAD, hPS19 and hPS-5X genotypes respectively, One way ANOVA.)

Supplementary Figure 2.

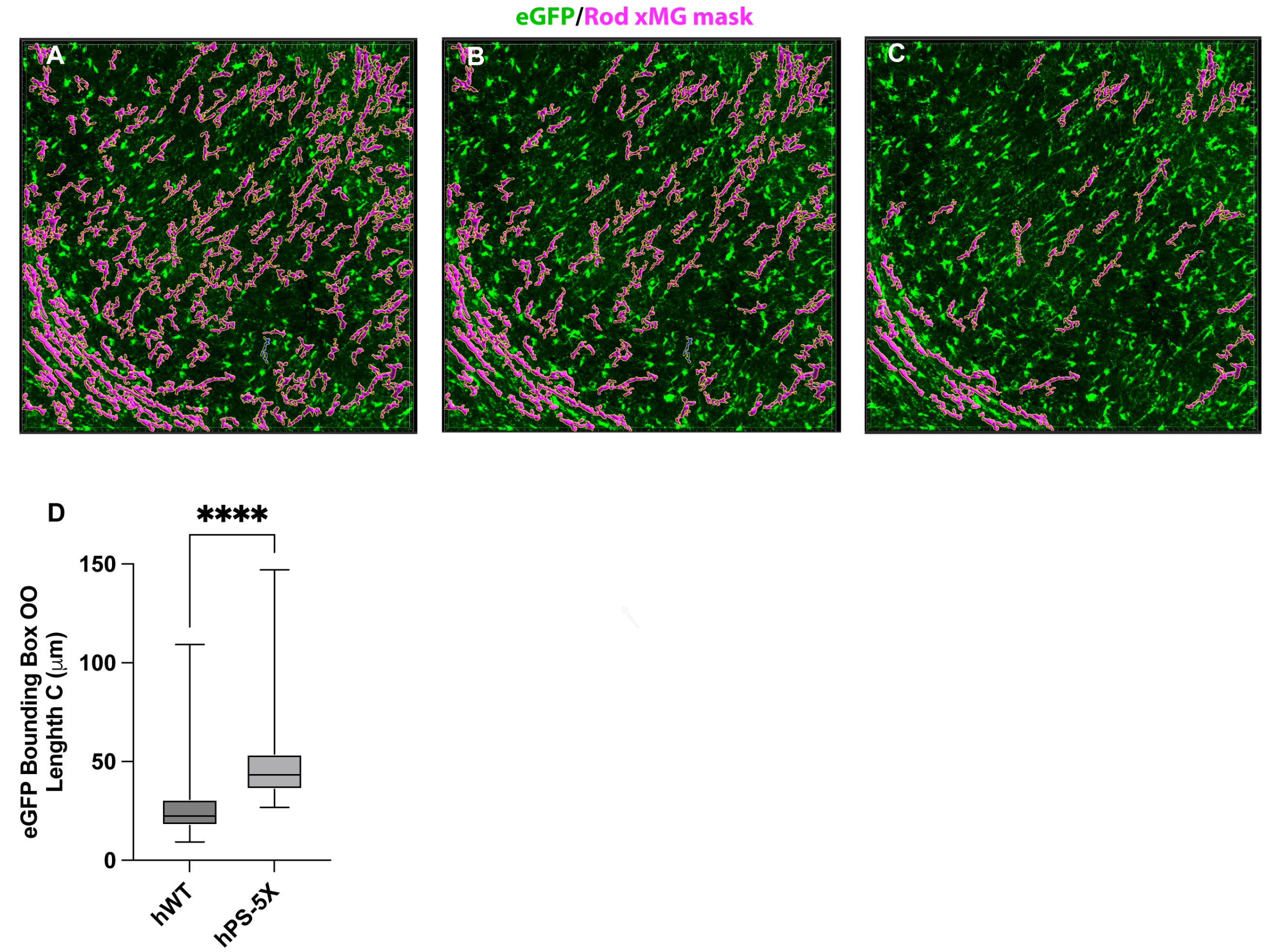

Suppl. Fig. 2. Analysis of rod human xMG (eGFP) by bounding box OO Length C in IMARIS software. Setting the cutoff length at (A) 40µm, (B) 50µm and (C) 60µm in IMARIS demonstrates that optimal identification of rod microglia (magenta mask) is achieved using a 50µm cutoff length. D) Quantification of the eGFP bounding Box OO Length C in hWT and hPS-5X mice reveals a significant difference between the average length of microglia in hWT mice versus hPS-5X mice (t-test, p-value< 0.0001).
